# *IL7RA* single nucleotide polymorphisms are associated with the size and function of the MAIT cell population in treated HIV-1 infection

**DOI:** 10.3389/fimmu.2022.985385

**Published:** 2022-10-20

**Authors:** Fei Han, Muhammad Yaaseen Gulam, Yichao Zheng, Nurul Syuhada Zulhaimi, Wan Rong Sia, Dan He, Amanda Ho, Leila Hadadi, Zhenyu Liu, Peiwu Qin, Peter E. Lobie, Adeeba Kamarulzaman, Lin-Fa Wang, Johan K. Sandberg, Sharon R. Lewin, Reena Rajasuriar, Edwin Leeansyah

**Affiliations:** ^1^ Institute of Biopharmaceutical and Health Engineering, Tsinghua Shenzhen International Graduate School, Tsinghua University, Shenzhen, China; ^2^ Precision Medicine and Healthcare Research Centre, Tsinghua-Berkeley Shenzhen Institute, Tsinghua University, Shenzhen, China; ^3^ Programme in Emerging Infectious Diseases, Duke-National University of Singapore Medical School, Singapore, Singapore; ^4^ Centre of Excellence for Research in AIDS (CERiA), University of Malaya, Kuala Lumpur, Malaysia; ^5^ Department of Medicine, University of Malaya, Kuala Lumpur, Malaysia; ^6^ Center for Infectious Medicine, Department of Medicine, Karolinska Institutet, Stockholm, Sweden; ^7^ Peter Doherty Institute for Infection and Immunity, Melbourne University, Victoria, Australia

**Keywords:** MAIT cells, HIV-1, IL-7, CD127 (IL7Ra) gene, antiretroviral (ARV) therapy

## Abstract

MAIT cells are persistently depleted and functionally exhausted in HIV-1-infected patients despite long-term combination antiretroviral therapy (cART). IL-7 treatment supports MAIT cell reconstitution *in vivo* HIV-1-infected individuals and rescues their functionality *in vitro*. Single-nucleotide polymorphisms (SNPs) of the *IL-7RA* gene modulate the levels of soluble(s)IL-7Rα (sCD127) levels and influence bioavailability of circulating IL-7. Here we evaluate the potential influence of *IL-7RA* polymorphisms on MAIT cell numbers and function in healthy control (HC) subjects and HIV-1-infected individuals on long-term cART. Our findings indicate that *IL-7RA* haplotype 2 (H2*T), defined as T-allele carriers at the tagging SNP rs6897932, affects the size of the peripheral blood MAIT cell pool, as well as their production of cytokines and cytolytic effector proteins in response to bacterial stimulation. H2*T carriers had lower sIL-7Rα levels and higher MAIT cell frequency with enhanced functionality linked to higher expression of MAIT cell-associated transcription factors. Despite an average of 7 years on suppressive cART, MAIT cell levels and function in HIV-1-infected individuals were still significantly lower than those of HC. Notably, we observed a significant correlation between MAIT cell levels and cART duration only in HIV-1-infected individuals carrying *IL-7RA* haplotype 2. Interestingly, treatment with sIL-7Rα *in vitro* suppressed IL-7-dependent MAIT cell proliferation and function following cognate stimulations. These observations suggest that sIL-7Rα levels may influence MAIT cell numbers and function *in vivo* by limiting IL-7 bioavailability to MAIT cells. Collectively, these observations suggest that *IL-7RA* polymorphisms may play a significant role in MAIT cell biology and influence MAIT cells recovery in HIV-1 infection. The potential links between *IL7RA* polymorphisms, MAIT cell immunobiology, and HIV-1 infection warrant further studies going forward.

## Introduction

Mucosa-associated invariant T (MAIT) cells are innate-like T cells and abundant in blood, the liver, and mucosal tissues (1). Classical MAIT cells in humans express an invariant T cell receptor (TCR) Vα7.2 coupled with restricted Jα 12/20/33 segments (2–4). In contrast to conventional T cells which recognize peptide fragments presented by the classical major histocompatibility complex (MHC) molecules, MAIT cells are activated by pyrimidine ligands bound to the non-polymorphic MHC-Ib related protein (MR1) (4–6). The TCR Vα7.2, together with high expression of CD161, IL-18 receptor α subunit (IL-18Rα), or recently used MR1 tetramers identify the MAIT cell population in humans (6–8). Although initially identified within the double negative (DN) T cell pool, peripheral blood MAIT cells predominantly express CD8 (9, 10). MAIT cells detect bacterial infection through metabolites of riboflavin biosynthetic pathway, such as 5-OP-RU, presented on MR1 (5). Additionally, MAIT cells can be activated in a TCR-independent manner by cytokines including Interleukin-1β (IL-1β), IL-12, IL-18, and Interferon-α (IFNα) (11, 12). In humans, MAIT cells display antimicrobial responsiveness in the intestinal mucosa pre-natally, prior to exposure to established commensal microflora (13).

Viruses do not directly give rise to MR1-presented riboflavin metabolite antigens. However, due to their high sensitivity to TCR-independent signals, mainly to IL-12 and IL-18, MAIT cells can produce IFNγ in response to different viruses, including influenza A, SARS-CoV-2, HBV/HDV, and HIV-1 (14–21). We and others have reported the loss of MAIT cells in circulation during chronic HIV-1 infection (19, 22–24). Residual MAIT cells from chronically HIV-1-infected patients are highly activated and show functional impairment in response to *Escherichia coli* stimulation (19, 23, 25). However, during the first weeks of HIV-1 infection, absolute counts of intestinal and circulating MAIT cells are elevated, indicating the proliferation of MAIT cells at the early phase of acute HIV-1 infection (24, 26). Various mechanisms of MAIT cell loss during chronic HIV-1 infection were recently reviewed by Han et al. (25), including cytokine dysregulation (27), persistent activation by microbial metabolites from microbial translocation (28, 29), MAIT cell death due to apoptosis and pyroptosis (30), and homing to inflamed tissues (23, 25). Unfortunately, combination antiretroviral therapy (cART) is ineffective in restoring the circulating MAIT cell pool (19, 24).

MAIT cells express high levels of IL-7Rα (CD127) (31, 32), and IL-7 promotes the activation of circulating MAIT cells, arms cytotoxic capacity, and enhances cytokine production (32). Furthermore, IL-7 amplifies the activation of MAIT cells *via* TCR or non-TCR signals (31, 32). In addition, IL-7 induces high levels of IL-18Rα on MAIT cells and increases their expression of the transcription factors promyelocytic leukemia zinc finger (PLZF), retinoic acid receptor-related orphan receptor (ROR)γt, T-box transcription factor 21 (TBX21, also known as T-bet), and eomesodermin (Eomes) (32–34). In disease settings, plasma IL-7 levels positively correlate with MAIT cell frequencies in HIV-1-infected patients (32, 35). IL-7 treatment expands the MAIT cell compartment in HIV-1-infected patients *in vivo* and rescues the antimicrobial effector function of MAIT cells of HIV-1-infected patients in response to *E. coli* stimulation *in vitro* (32, 36). In rhesus macaques with chronic SHIV infection, IL-7 alone enhances the proliferation but not degranulation of MAIT cells (33). In mice, pretreatment with IL-7 improves survival in *Streptococcus pneumoniae* infection (34).

IL-7 is a homeostatic cytokine required for both the production and survival of naïve and memory T cells and is critical for immune recovery in lymphopenic states (37, 38). IL-7 exerts its biological effect by binding to its membrane-bound receptor, mIL-7Rα on T-cells (39). There is also a soluble form of this receptor (sIL-7Rα), which is thought to modulate IL-7 availability (40). *In vitro* studies found that levels of sIL-7Rα in culture affected IL-7-mediated functions including T cell survival and proliferation (41). This suggests that sIL-7Rα may influence IL-7 mediated function including its capacity to drive immune recovery in HIV-1-infected individuals.

Four common haplotypes of the *IL7RA* gene (haplotypes 1, 2, 3 and 4) have been previously defined and shown to be associated with sIL-7Rα levels (42, 43). Haplotype 2 (tagged by carriage of T allele in rs6897932) is associated with reduced sIL7Rα levels in plasma. This single nucleotide polymorphism (SNP) influences the amount of soluble and membrane bound IL-7Rα isoforms produced by disrupting an exon splicing silencer (44). Studies assessing the association between rs6897932 and immune reconstitution have reported both positive and negative associations. In a predominantly Caucasian Australian cohort of HIV-1-infected people, there was a significant association between the *IL7RA* haplotype 2 (H2*T) and faster CD4^+^ T cell recovery in those receiving suppressive ART (45). In a replicate study in Uganda, the *IL7RA* H2*T had an inverse association with CD4^+^ T cell recovery compared to that found in Caucasians, and was associated with poorer CD4^+^ T cell recovery (46). Subsequently, the differential influences of *IL7RA* haplotypes on HIV-1 disease progression in Caucasians and Africans have been confirmed (47, 48). The reason for the different associations seen in the various ethnic populations despite a consistent influence of *IL7RA* haplotype 2 on low sIL-7Rα levels is currently unclear. T-allele carriers of the rs6897932 SNP have also been associated with reduced susceptibility to multiple autoimmune diseases including multiple sclerosis, systemic lupus erythematosus (49), chronic inflammatory arthropathies (50), sarcoidosis (51) and type 1 diabetes (52) in HIV-1-uninfected individuals. Despite the wide ranging effects of this SNP in multiple immune-mediated diseases, there is a notable lack of studies systematically assessing its immunologic effects.

Although the effects of IL-7 on MAIT cells have been clear for some time, it is unknown whether *IL7RA* polymorphisms affect MAIT cell phenotype and function in health and during HIV-1 infection. In this study, we investigate the association of *IL7RA* haplotypes with MAIT cell levels and effector function in health and in chronic HIV-1 infection with long-term cART-mediated viral suppression. Our findings indicate that *IL7RA* H2*T affects the size of the peripheral MAIT cell pool, as well as their production of cytokines and cytolytic effector proteins in response to bacterial stimulation. Furthermore, presence of *IL7RA* H2*T positively associates with circulating MAIT cell levels following the long-term suppressive ART in chronically HIV-1-infected individuals.

## Results

### *IL7RA* haplotype 2 is associated with higher MAIT cell levels in healthy individuals

**Figure 1:**
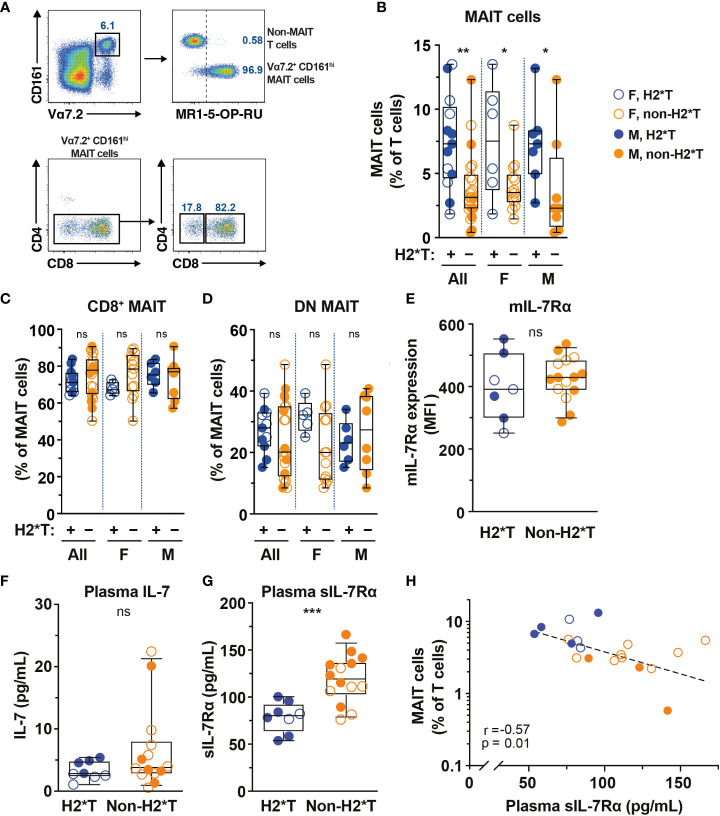
MAIT cell levels are higher in HIV-1-uninfected individuals carrying the *IL7RA* haplotype 2. **(A)** Representative flow cytometry gating strategy to identify MAIT cells by TCR Vα7.2 and CD161 co-expression on T cells (Vα7.2^+^ CD161^hi^ MAIT cells), as well as the CD4 and CD8 co-receptor expression. MR1-5-OP-RU tetramer staining was used to confirm the identity and MR1-restriction of Vα7.2^+^ CD161^hi^ MAIT cells. **(B–E)** MAIT cell levels **(B)**, the distribution of CD8^+^ **(C)** and CD4^-^ CD8^-^ (DN; **D**) MAIT cell subsets, and membrane-bound (m)IL-7Rα **I** levels by MAIT cells in HIV-1-uninfected individuals bearing the different IL-7Rα haplotypes. **(F, G)** Plasma levels of IL-7 **(F)** and soluble (s)IL-7Rα **(G)** in HIV-1-uninfected individuals bearing the different IL-7Rα haplotypes. **(H)** Spearman’s correlation between circulating MAIT cell levels and plasma sIL-7Rα. Box and whisker plots show all data points (N=7-13 for haplotype 2 individuals and N=14-22 for non-haplotype 2), median, and the interquartile range. Statistical significance was determined using Mann-Whitney’s test **(B–F)** or unpaired t-test **(G)**. Correlations were assessed using the Spearman rank correlation **(H)**. *** p<0.001, ** p<0.01,* p<0.05. ns, not significant. F, female; M, male; H2*T, *IL7RA* haplotype 2.

IL-7 treatment supports increased MAIT cell numbers *in vivo* in humans and *IL7RA* SNPs influence IL-7 uptake and downstream biological activity (36, 53). Thus, the relationship between *IL7RA* SNPs (**Supplementary Table 1**) and MAIT cell numbers in circulation of healthy individuals (**Supplementary Table 2**) was initially determined. Peripheral blood (PB) MAIT cells were identified by the combined staining of TCR Vα7.2 with high expression of CD161 (**Figure 1A**). The vast majority of Vα7.2^+^ CD161^hi^ MAIT cells also stained positive with MR1-5-OP-RU tetramer and expressed the CD8α co-receptor (**Figure 1A**), consistent with previously published studies (10, 54). Healthy individuals carrying *IL7RA* H2*T, defined as T-allele carriers (heterozygous or homozygous) at the tagging SNP rs6897932 (**Supplementary Table 1**), had higher levels of MAIT cells in circulation than those with non-haplotype 2 (non-H2*T) polymorphisms (**Figure 1B**). There was no significant age differences between the haplotype groups when stratified by sex (**Supplementary Figure 1A**). Furthermore, MAIT cell numbers were comparable between females and males when stratified by haplotypes (**Supplementary Figure 1B**). Correlative analyses also showed that there were no associations between age and MAIT cell numbers as a whole or stratified either by sex or haplotypes (**Supplementary Table 3**). Therefore, the difference in MAIT cell frequency between the *IL7RA* haplotypes was probably not influenced by differences in age or sex between the groups. There was no difference in the representation of CD8^+^ and DN MAIT cells between the *IL7RA* haplotypes as a whole or when stratified by sex (**Figures 1C, D**). Similarly, no differences were observed in IL-7Rα surface expression (mIL-7Rα/CD127) by MAIT cells (**Figure 1E**) and the plasma levels of IL-7 from individuals carrying different *IL7RA* haplotypes (**Figure 1F**), but those with H2*T haplotypes had significantly lower sIL-7Rα in their plasma (**Figure 1G**), consistent with previous studies (42, 44). Notably, there was a significant inverse correlation between MAIT cell frequency and plasma levels of sIL-7Rα in this cohort of healthy individuals (**Figure 1H**). However, there was no correlation between age and plasma levels of sIL-7Rα as a whole or stratified by haplotype or sex (**Supplementary Table 3**). Altogether, these results suggest that *IL7RA* polymorphisms associates with MAIT cell levels in healthy adults.

### *IL7RA* SNPs are associated with MAIT cell expression of transcription factors and function in healthy individuals

**Figure 2:**
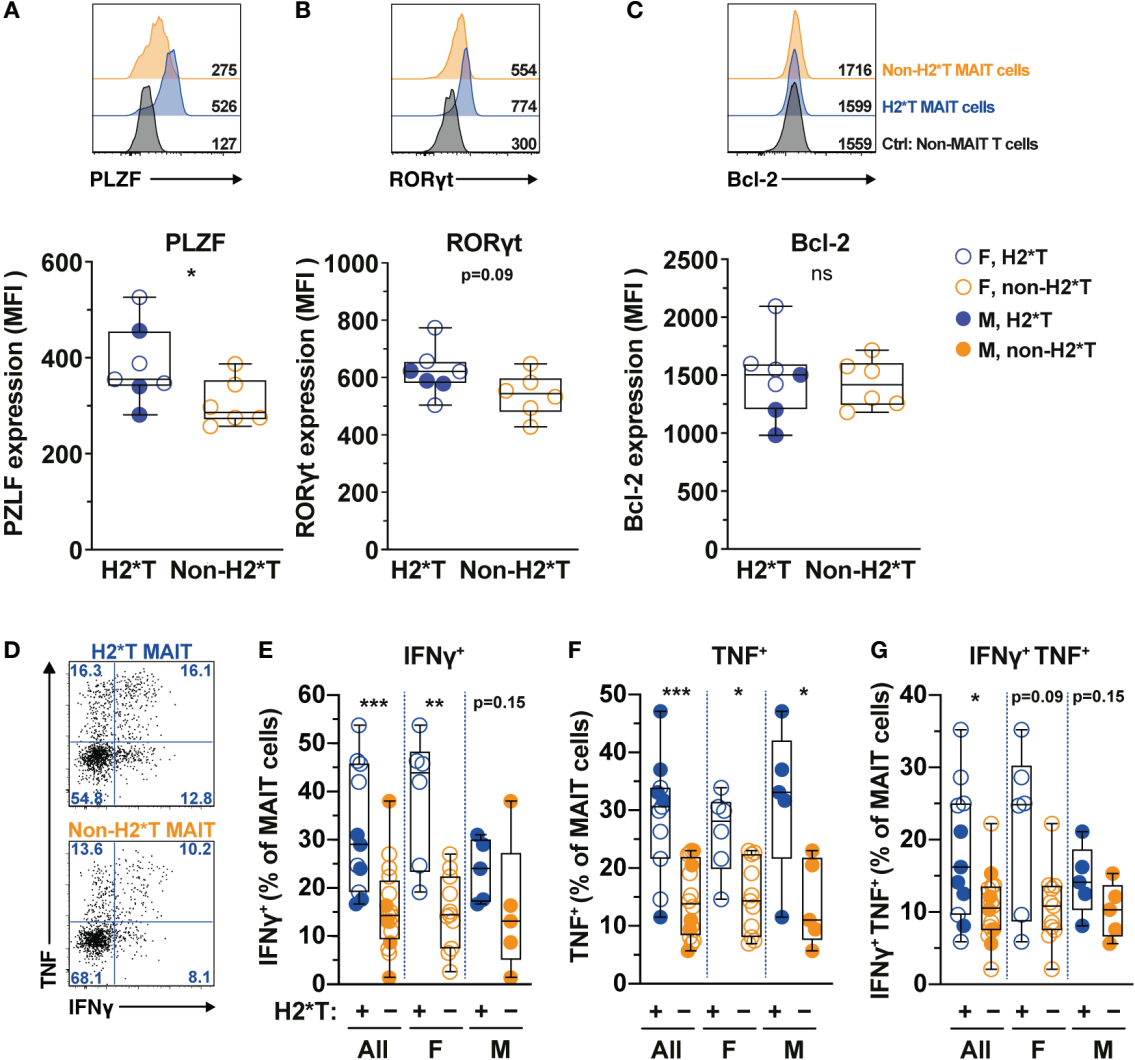
MAIT cells from HIV-1-uninfected individuals carrying *IL7RA* haplotype 2 express higher levels of MAIT cells-associated transcription factors and effector function. **(A–C)** Representative histograms for PLZF **(A)**, RORγt **(B)**, and Bcl-2 **(C)** levels in Vα7.2^+^ CD161^hi^ MAIT cells in HIV-1-uninfected individuals bearing the different IL-7Rα haplotypes. Non-MAIT T cells represent all other non-MAIT, CD3^+^ T cells **(D–G)** Representative flow cytometry gating strategy **(D)** to enumerate IFNγ **(E)**, TNF **(F)**, and dual IFNγ-TNF **(G)** production by MAIT cells following 24 h stimulation with formaldehyde-fixed *E coli* (bacterial dose 1 CFU/cell). Box and whisker plots show all data points (N=7-11 for haplotype 2 individuals and N=6-16 for non-haplotype 2), median, and the interquartile range. Statistical significance was determined using Mann-Whitney’s test. *** p<0.001, ** p<0.01,* p<0.05. ns, not significant. F, female; M, male; H2*T, *IL7RA* haplotype 2.

MAIT cell treatment with IL-7 *in vitro* leads to enhanced expression of transcription factors (TFs) and augmented MAIT cell antimicrobial function, such as increased production of IFNγ and TNF (31, 32). Therefore, whether *IL7RA* polymorphisms affect expression of critical TFs and MAIT cell function was next evaluated. In line with higher MAIT cell frequency in individuals carrying H2*T, MAIT cells in these individuals expressed higher levels of PLZF (**Figure 2A**). RORγt expression also appeared to be higher in MAIT cells from H2*T individuals (**Figure 2B**). IL-7 downstream biological activity is associated with increased cellular survival partly *via* the anti-apoptotic protein Bcl-2 (55). However, Bcl-2 expression levels in MAIT cells from individuals carrying the H2*T and non-H2*T haplotypes were comparable (**Figure 2C**). We were unable to stratify these differences based on sex due to limited sample size. To assess for antimicrobial effector function, MAIT cells were activated using an assay that was previously developed by our group (56, 57). Following stimulation with formaldehyde-fixed *E. coli* for 24 h, MAIT cells from healthy adults with the H2*T haplotype had increased levels of IFNγ and TNF, and appeared to also be more bifunctional (**Figures 2D–G**). Such higher expression levels of cytokines were still evident in females after stratification by sex, whereas there was a trend of increasing cytokine production by MAIT cells from male H2*T *IL7RA* carriers. There was no correlation between age and cytokine expression by MAIT cells stratified by either sex or haplotype (**Supplementary Table 3**). These findings suggest that *IL7RA* polymorphism may affect MAIT cell functional activity *in vivo* through modulation of TF levels.

### MAIT cell levels and function are significantly lower in HIV-1-infected individuals on long-term suppressive ART

**Figure 3:**
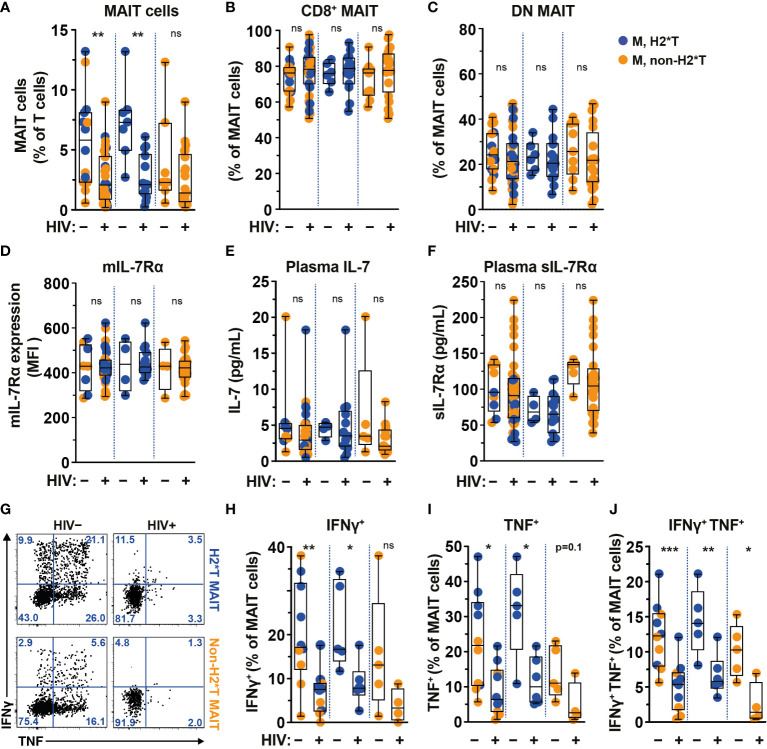
Comparison of MAIT cells in HIV-1-uninfected and -infected individuals with long-term viral suppression and their relationship with the *IL7RA* SNP. **(A–E)** Vα7.2^+^ CD161^hi^ MAIT cell levels **(A)**, the distribution of CD8^+^ **(B)** and CD4^-^ CD8^-^ (DN; **C**) MAIT cell subsets, and membrane-bound (m)IL-7Rα **(D)** levels by MAIT cells in HIV-1-uninfected and HIV-1-infected individuals carrying the different *IL7RA* haplotypes. **(E, F)** Plasma levels of IL-7 **(E)** and soluble (s)IL-7Rα **(F)** in HIV-1-uninfected and HIV-1-infected individuals individuals bearing the different *IL7RA* haplotypes. **(G–J)** Representative flow cytometry gating strategy **(G)** to enumerate IFNγ **(H)**, TNF **(I)**, and dual IFNγ-TNF **(J)** production by MAIT cells following 24 h stimulation with formaldehyde-fixed *E coli* (bacterial dose 1 CFU/cell). Box and whisker plots show all data points (**A–F**: HIV-1-uninfected N=8-14, N=4-7 for both haplotype 2 and non-haplotype 2; HIV-1-infected N=34-39, N=14-15 for haplotype 2 and N=20-24 for non-haplotype 2; **H–J**: HIV-1-uninfected N=10, N=5 for both haplotype 2 and non-haplotype 2; HIV-1-infected N=10, N=6 for haplotype 2 and N=4 for non-haplotype 2), median, and the interquartile range. Statistical significance was determined using Mann-Whitney’s test. *** p<0.001,** p<0.01,* p<0.05. ns, not significant. M, male; H2*T, *IL7RA* haplotype 2. Male HC dataset were retrieved from HC cohort depicted in **Figure 1**.

The frequency and function of MAIT cells are impaired in chronic untreated HIV-1-infected patients and do not fully recover following long-term cART (19, 22, 25). In agreement with previous studies (19, 22, 58), circulating MAIT cell levels from HIV-1-infected individuals with long-term cART (**Supplementary Table 4**) were still significantly lower than those from uninfected adult males from the same HC cohort (**Figure 3A**). The persistent depletion of MAIT cells from the circulation of long-term treated HIV-1-infected individuals was still observed in IL-7RA haplotype 2 carriers but not in non-haplotype 2 carriers (**Figure 3A**). Since there was no association between MAIT cell levels and age stratified by haplotype in uninfected and HIV-1-infected cohorts in the current study (**Supplementary Tables 3**, **5**, respectively), the differences seen in MAIT cell levels may be less likely due to age differences between the HC and HIV-1-infected cohorts. Similarly, no differences in the proportion of CD8^+^ and DN MAIT cell subpopulations, as well as in the expression of IL-7Rα by MAIT cells, were noted in healthy controls as compared with those of HIV-1-infected patients (**Figures 3B-D**). There was no difference in plasma levels of IL-7 and sIL-7Rα between healthy controls and HIV-1-infected patients bearing H2*T and non-H2*T haplotypes (**Figures 3E, F**). In addition, HIV-1 infection was associated with poor IFNγ and TNF production by MAIT cells following *E. coli* stimulation despite long-term cART (**Figures 3G–J**) in line with previous observations (19). This impairment was more obvious in HIV-1-infected individuals carrying the non-H2*T haplotypes (**Figures 3G–J**). The low sample size for HIV-1-infected individuals carrying the non-H2*T haplotype may explain why there was no significant difference in IFNγ expression found when compared to healthy controls carrying the same *IL7RA* haplotype. Taken together, these results indicate that MAIT cell dysfunction in chronic HIV-1 infection persists despite long-term viral suppression when compared to healthy controls. This dysfunction may occur regardless of the *IL7RA* haplotypes.

### *IL7RA* haplotypes associate with MAIT cell function and recovery in HIV-1-infected patients on ART

**Figure 4:**
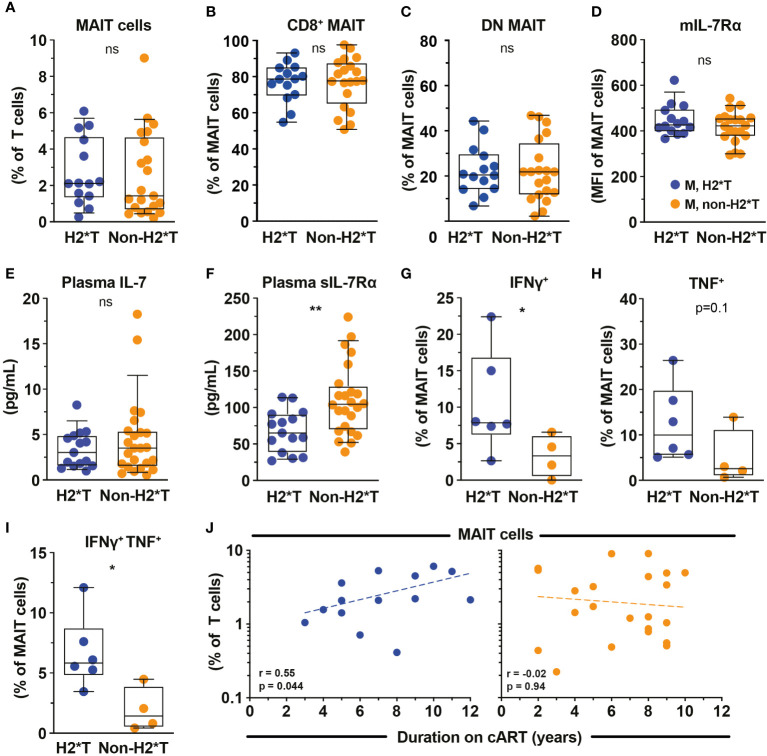
MAIT cell levels and functions in HIV-1-infected individuals with long-term viral suppression and their relationship with the IL-7Rα SNP. Vα7.2^+^ CD161^hi^ MAIT cell levels **(A)**, the distribution of CD8^+^ **(B)** and CD4^-^ CD8^-^ (DN; **C)** MAIT cell subsets, and membrane-bound (m)IL-7Rα **(D)** by MAIT cells in HIV-1-infected individuals bearing the different *IL7RA* haplotypes. **(E, F)** Plasma levels of IL-7 **(E)** and soluble (s)IL-7Rα **(F)** in HIV-1-infected individuals bearing the different *IL-7RA* haplotypes. **(G–I)** The IFNγ **(G)**, TNF **(H)**, and dual IFNγ-TNF **(I)** production by MAIT cells following 24 h stimulation with formaldehyde-fixed *E coli* (bacterial dose 1 CFU/cell). **(J)** Spearman’s correlation between circulating MAIT cell levels and duration of cART in individuals bearing haplotype 2 (N=14) and non-haplotype 2 (N=22). Box and whisker plots show all data points (**A–E**: N=14-15 for haplotype 2 and N=20-24 for non-haplotype 2; **G–I**: N=6 for haplotype 2 and N=4 for non-haplotype 2), median, and the interquartile range. Statistical significance was determined using Mann-Whitney’s test. ** p<0.01,* p<0.05. ns, not significant. M, male; H2*T, *IL7RA* haplotype 2. HIV-1-infected dataset were retrieved from HIV-1-infected cohort depicted in **Figure 3**.

Based on the findings that MAIT cell dysfunction persists in HIV-1-infected patients receiving long-term cART, we next revisited the dataset from the same HIV-1-infected cohort and evaluated the degree of impairment between patients carrying the H2*T and non-H2*T haplotypes. No significant differences in clinical parameters were noted between the HIV-1-infected patient groups carrying *IL7RA* haplotypes H2*T and non-H2*T, including age, pre-treatment CD4 counts and viral loads, duration of ART (**Supplementary Table 4**). Interestingly, there was comparable MAIT cell frequency in virally-suppressed chronic HIV-1-infected patients with H2*T and non-H2*T haplotypes (**Figure 4A**). Additionally, haplotypes H2*T and non-H2*T did not appear to influence MAIT cell major subset distribution within the HIV-1-infected patient group (**Figures 4B, C**). Although comparable levels mIL-7Rα and plasma IL-7 were observed between the two groups (**Figures 4D, E**), individuals carrying non-H2*T haplotype had a higher level of plasma sIL-7Rα (**Figure 4F**), consistent with the observation in healthy individuals (**Figure 1G**). Moreover, there was no association between age and plasma sIL-7Rα levels as a whole or stratified by haplotype in this HIV-1-infected cohort (**Supplementary Table 5**). In addition, HIV-1-infected individuals with H2*T haplotype had better MAIT cell functionality following bacterial stimulation when compared to those carrying other haplotypes (**Figures 4G–I**). There was also no correlation between age and cytokine expression by MAIT cells following *E. coli* stimulation as a whole or stratified by haplotype in the current HIV-1-infected cohort, although the sample size was small (**Supplementary Table 5**). Interestingly, despite that there was no difference in MAIT cell levels between group with the different *IL7RA* haplotypes, duration of ART positively correlated with MAIT cell levels in individuals bearing the H2*T haplotype (**Figure 4J**).

### Soluble IL-7Rα inhibits IL-7-mediated MAIT cell proliferation and effector function *in vitro*

**Figure 5:**
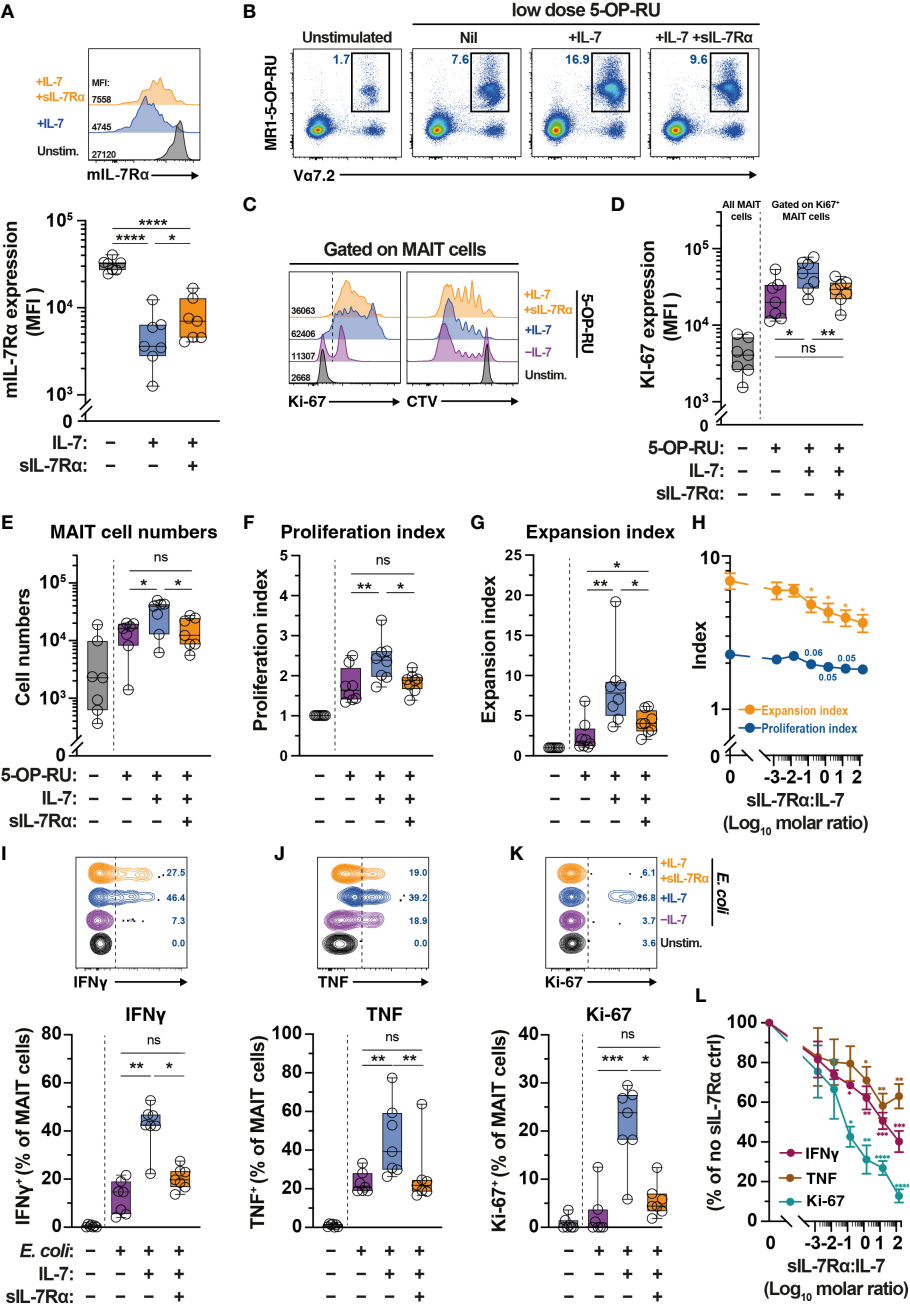
Soluble IL-7Rα inhibits IL-7-mediated MAIT cell proliferation and effector function. **(A)** Expression levels of mIL-7Rα on MR1-5-OP-RU tetramer^+^ Vα7.2^+^ MAIT cells following 24 h treatment of PBMC with IL-7 alone or together with sIL-7Rα (molar ratio 1:1) (N=7). **(B–G)** MR1-5-OP-RU tetramer^+^ Vα7.2^+^ MAIT cell **(B)** proliferation as determined by Ki-67 expression levels on Ki-67^+^ MAIT cells **(C**; *left panel***, D)**, absolute MAIT cell numbers **(E)**, and proliferation **(F)** and expansion **(G)** indices calculated using CTV-dilution **(C**; *right panel***)** following 5-days treatment of PBMC with 5-OP-RU, 5-OP-RU+IL-7, or 5-OP-RU+IL-7+sIL-7Rα (sIL-7Rα/IL-7molar ratio 1:1) (N=7-8). **(H)** Dose-dependent effect of sIL-7Rα treatment on MAIT cells proliferation and expansion indices following 5-days treatment with IL-7- and 5-OP-RU (N=6-7). **(I-K)** MAIT cell expression of IFNγ **(I)**, TNF **(J)**, and Ki-67 **(K)** after 24 h treatment of PBMC with IL-7 alone or together with sIL-7Rα (molar ratio 1:1) followed by a further 24 h stimulation with formaldehyde-fixed *E coli* (bacterial dose 0.1 CFU/cell) (N=7). **(L)** Dose-dependent effect of sIL-7Rα treatment on MAIT cells cytokine and Ki-67 expression following treatment with *E coli* with or without IL-7 priming as in **(I–K)** (N=6-7). Expression levels for **(L)** were normalised to nil sIL-7Rα controls to minimise inherent donor-to-donor variability, with statistical tests were performed on raw data. Box and whisker plots show all data points, median, and the interquartile range, whereas data presented as line graphs with error bars represent the mean and standard error. Statistical significance was determined using the Friedman test followed by Dunn’s *post-hoc* test **(E, I, J, K)**, or repeated measure one-way ANOVA with Tukey’s *post-hoc* test **(A, D, F,G)**, or mixed-effect analysis with Dunnett’s *post-hoc* test **(H, L)**. **** p<0.0001, *** p<0.001, ** p<0.01, * p<0.05, ns, not significant.

Levels of sIL-7Rα affects IL-7-mediated functions including T cell survival and proliferation (41). Given plasma sIL-7Rα levels negatively correlate with MAIT cell frequency (**Figure 1H**), we next explored the impact of sIL-7Rα treatment *in vitro* on IL-7-mediated MAIT cell proliferation and function. Downregulation of surface expression of mIL-7Rα is a surrogate marker of MAIT cell activation following IL-7 treatment *in vitro* (32). By using this approach, initial experiments showed that sIL-7Rα mitigated the downregulation of mIL-7Rα on MAIT cells following IL-7 treatment *in vitro* (**Figure 5A**), suggesting that sIL-7Rα reduces IL-7 binding to mIL-7Rα on MAIT cells. Indeed, sIL-7Rα treatment *in vitro* reduced MAIT cell proliferation following stimulation with low dose of the prototypical MR1 ligand 5-OP-RU supplemented with low dose IL-7 (Figs. 5B-5G). This was shown by lower levels of Ki67 expression, lower MAIT cell numbers, as well as reduced proliferation and expansion indices calculated from CellTrace Violet (CTV) dilution assay (**Figures 5B–G**), performed as previously described (56, 59). Such inhibition appeared to be dose-dependent and remained apparent at sIL-7Rα:IL-7 molar ratio > 100 (**Figure 5H**). The inhibitory effect of sIL-7Rα on MAIT cell proliferation did not occur when IL-2 substituted IL-7 (**Supplementary Figures 2A–G**), indicating that sIL-7Rα-mediated impairment is specific to IL-7-dependent activity on MAIT cells. Interestingly, sIL-7Rα also inhibited IL-7-mediated enhancement on resting MAIT cell IFNγ and TNF production and Ki-67 expression following *E. coli* stimulation (**Figures 5I–K**). This inhibition was also dose-dependent as MAIT cell cytokine production and Ki-67 expression remained reduced at sIL-7Rα:IL-7 molar ratio > 100 (**Figure 5L**).

## Discussion

In the present study, we report an association between *IL7RA* polymorphisms and MAIT cell levels and functional activity in healthy individuals and HIV-1-infected individuals with long-term viral suppression. Specifically, we show that *IL7RA* haplotype 2 positively impacts the frequency and functional activity of MAIT cells in healthy individuals. This *IL7RA* haplotype is correlated with decreased plasma sIL-7Rα concentration both in health and chronic HIV-1 infection, which is linked with higher MAIT cell levels in healthy controls. Importantly, MAIT cell levels correlate with duration on cART in HIV-1-infected individuals carrying *IL7RA* haplotype 2, suggesting favorable MAIT cell reconstitution in these individuals. Such MAIT cells also appear to display better functionality upon bacterial stimulation. This observation suggests that *IL7RA* haplotype 2 may be beneficial for MAIT cell reconstitution *in vivo*. Finally, *in vitro* experiments support the notion that the positive effects of *IL7RA* haplotype 2 on MAIT cell number and function is associated with lower sIL-7Rα concentration, as sIL-7Rα treatment *in vitro* effectively inhibits IL-7-dependent MAIT cell proliferation and effector function. Altogether, these observations suggest favorable effects of the *IL7RA* haplotype 2 on MAIT cell reconstitution in virally suppressed chronic HIV-1 infection.

How *IL7RA* polymorphisms may affect MAIT cell biology in health and during HIV-1-infection is unclear. It is possible that decreased production of sIL-7Rα isoform in *IL7RA* haplotype 2-carrying healthy and HIV-1-infected individuals may diminish the interference with IL-7 binding to its membrane-bound receptors on T cells (42, 53). However, the effects of sIL-7Rα in shaping IL-7 bioavailability are complex. Several reports demonstrated that sIL-7Rα serves as a reservoir of IL-7 and hence increases its half-life due to relatively low affinity (53, 60). Lundström et al. provided a model for sIL-7Rα-mediated IL-7 regulation, proposing that sIL-7Rα/rhIL-7 with a very high molar ratio (>5000) may increase the bioactivity of IL-7 by stabilizing its reservoir (53). Furthermore, *in vivo* experiments showed that IL7^−/−^ mice had transiently increased IL-7 levels following administration of rhIL-7 with sIL-7Rα versus rhIL-7 alone (53). In the current study, we saw the plasma levels of sIL-7Rα were significantly lower in haplotype 2 individuals in both healthy and HIV-1-infected individuals, consistent with previous studies (42, 44). Moreover, we found that MAIT cell levels in *IL7RA* haplotype 2 carriers with chronic HIV-1 infection correlated with duration on cART, whereas non-haplotype 2 carriers did not show this correlation. This observation is in line with better recovery from lymphopenia for haplotype 2 carriers on cART (42). HIV-1-infected *IL7RA* haplotype 2 carriers also had partial recovery of the cytokine production following *E. coli* stimulation. Given the stimulatory effects of IL-7 on MAIT cells (31, 32, 61), all these together suggest that *IL7RA* haplotype 2 may influence MAIT cell homeostasis *via* enhanced bioactivity of IL-7 due to low plasma sIL-7Rα.

Indeed, our data indicate dose-dependent inhibition of IL-7-mediated MAIT cell proliferation and effector function by sIL-7Rα *in vitro*. Firstly, sIL-7Rα mitigated the downregulation of mIL-7Rα following low dose IL-7, indicating reduced binding of IL-7 to mIL-7Rα on MAIT cells. Furthermore, sIL-7Rα-mediated inhibition was seen when MAIT cells were stimulated with low dose of the prototypical antigen 5-OP-RU in the presence of low dose of IL-7, but not when in the presence of low dose of a similar γc cytokine IL-2. This indicates the specificity of sIL-7Rα-mediated inhibition on IL-7. Finally, sIL-7Rα negates IL-7-dependent enhancement of MAIT cell effector function following *E. coli* stimulation. Particularly, IFNγ and TNF levels by IL-7-primed MAIT cells were comparable to those of unprimed MAIT cells following *E. coli* stimulation when co-administered with sIL-7Rα. Collectively, our *in vitro* data support the notion that favorable effects of *IL7RA* haplotype 2 on MAIT cell numbers and function in both HC and HIV-1-infected patients is at least partially a consequence of enhanced bioavailability of IL-7 to MAIT cells due to lower sIL-7Rα levels *in vivo*. Our *in vitro* study used much lower sIL-7Rα/rhIL-7 molar ratios (0.015 to 150) and showed inhibitory effect of sIL-7Rα on IL-7 bioactivity, whereas a previous study that used a very high sIL-7Rα/rhIL-7 molar ratio of 5000 showed an enhancement of IL-7 bioactivity (53). Therefore, because of the complexity of IL-7/IL-7Rα biology, it will be important to further examine the influence of these receptor polymorphisms on IL-7 utilization and MAIT cell biology and the mechanisms behind these effects in both HC and HIV-1-infected individuals going forward.

The positive effects of haplotype 2 on MAIT cells may additionally occur through the modulation of critical TFs for MAIT cell functions. Our data indicate that the *IL7RA* haplotype 2 appears to support higher expression of PLZF and RORγt by peripheral MAIT cells in healthy individuals. These TFs are essential for both TCR-dependent and -independent MAIT cell development and cytokine production (3). Therefore, upregulation of PLZF and RORγt may potentially lead to higher frequency and function of MAIT cells in haplotype 2 individuals. Tang et al. observed upregulation of anti-apoptotic molecule Bcl-2 in intrasinusoidal MAIT cells upon IL-7 stimulation (31). Of note, sIL-7Rα inhibits IL-7–induced proliferation and Bcl-2 expression in peripheral CD8^+^ T cells (41, 53). Thus, lower sIL-7Rα and increased IL-7 utilisation by MAIT cells in haplotype 2 individuals may potentially lead to enhanced survival of MAIT cells *via* increased Bcl-2 activity. However, in the current study we did not observe any changes in Bcl-2 expression in the setting of lower plasma sIL-7Rα levels in haplotype 2 individuals.

Our study has several important limitations. Firstly, our sample size was relatively small and there was some differences with regard to age and sex distribution between the healthy and HIV-1-infected cohorts. Nevertheless, our correlative analyses suggest that these differences do not seem to significantly associate with the MAIT cell levels in either HC or HIV-1-infected individuals. Indeed, the differences in MAIT cell numbers and function in individuals carrying different *IL7RA* haplotypes hold when stratified by sex in HC cohort. Unfortunately, the low number of male donors from either haplotypes in HC cohort does not allow us to make firm conclusion on the influence of *IL7RA* haplotypes on MAIT cell biology in adult males. We also used only the dataset from male HC for the comparison with the all-male HIV-1-infected cohort, thereby removing biological sex as a confounding factor in these analyses. Secondly, the potential influence of other confounders cannot be ruled out due to the relatively small sample size. In future studies, it will be important to recruit a larger cohort with matching healthy controls, as well as longitudinal cohorts from the HIV-1-infected individuals to confirm better MAIT cell recovery of haplotype 2 carriers. Moreover, further *in vitro* studies are warranted to tease out the potential mechanisms by which IL-7 and *IL-7RA* haplotypes influence MAIT cell biology. Complex interaction between MAIT cell biology, *IL-7RA* polymorphisms, and HIV-1 infection should be explored in-depth going forward. Understanding intricate utilization of IL-7 in reconstituting MAIT cells and enhancing their functions may ultimately benefit the host defense during HIV-1 infection.

## Resource availability

### Lead contact

Further information and requests for resources and reagents should be directed to and will be fulfilled by the Lead Contact, Edwin Leeansyah (https://edwin.leeansyah@sz.tsinghua.edu.cn)

### Materials availability

This study did not generate new unique reagents. The MR1 ligand 5-(2-oxopropylideneamino)-6-D-ribitylaminouracil (5-OP-RU) was kindly provided by Drs. David P. Fairlie and Jeffrey Y. W. Mak (University of Queensland, Australia) (62, 63).

### Data and code availability

No data sets or code were generated or analysed in this study. All raw data associated with this study are available upon request from the Lead Contact. All software is commercially available.

## Materials and methods

### Ethics statement

Human samples were collected after informed written consent was obtained from all donors in accordance with study protocols conforming to the provisions of the Declaration of Helsinki. All participants (HIV-1-infected and healthy volunteers) provided written informed consent and this study was approved by the University Malaya Medical Centre’s ethics review board (MEC Ref: 896.32) and the Tsinghua Shenzhen International Graduate School Ethics Committee (Ref: 202171).

### HIV-1-infected and uninfected cohorts

HIV-1-infected (n=39) individuals previously genotyped for the IL-7Rα SNPs were identified from a previous cohort established to explore biological factors associated with immune recovery (64). Briefly, all patients were recruited at the Infectious Diseases Unit (IDU) in University Malaya Medical Centre (UMMC) during their routine follow-up. Patients who fulfilled the following inclusion criteria were enrolled into the study: 1) Men or women aged at least 18 years, 2) first antiretroviral regimen composed of cART defined by at least three antiretroviral drugs, 3) CD4 T-cell count <500 cells/µl at the start of cART and 4) patients achieving HIV-1 RNA levels ≤50 copies/ml within 12 months following commencement of cART. Demographic and clinical parameters including age at cART initiation, gender, ethnicity, history of AIDS defining illnesses, antiretroviral prescribed and CD4 T cell count and HIV RNA measurements were obtained from patients medical records. HIV-1-negative controls (n=35) were recruited amongst healthy laboratory staff volunteers. All samples were processed using identical protocols within 4 hours of venipuncture.

### Blood processing

Peripheral blood mononuclear cells (PBMCs) were isolated by standard Ficoll-Histopaque density gradient separation (Axis-Shield Poc As™). Isolated PBMCs were cryopreserved in liquid nitrogen until further required.

### Flow cytometry analysis

Anti-Bcl-2 PE, CD3-FITC, CD8-APC, CD127-BV510, IFNγ-APC, RORγt-BV421, TNF-PE were from BD Biosciences. Anti-CD4-PerCP, CD8-BV510, CD127-PECy7, CD161-BV421, -FITC, and -PE, IFNγ-APC, Ki-67-AF488 and -BV510, TNF-BV421, and Vα7.2 PECy7 were from Biolegend. Anti-PLZF APC was from R&D Systems. CellTrace Violet (CTV) cell proliferation kit, Live/Dead Aqua and Near Infrared fixable cell stain were from Life Technologies. MR1-5-OP-RU tetramer -APC, -BV421, and -PE were from NIH Tetramer Core Facility, Emory University. Staining with the MR1 5-OP-RU tetramers was performed for 40 min at room temperature (RT) (6) before proceeding to the surface and intracellular staining with other mAbs. Cell surface and intracellular staining for TFs, cytokines, and cytotoxic molecules were performed as previously described (10). Samples were acquired on an FACS Canto II (BD Biosciences) equipped with 405, 488, and 633 nm lasers or CytoFLEX (Beckman Coulter) equipped with 405, 488, and 638 nm lasers. Data including the compensation platform were analysed using FlowJo v.10 (BD Biosciences).

### Preparation of MAIT cell antigen 5-OP-RU

The MAIT cell antigen 5-OP-RU was synthesized according to a previously published procedure (62, 63). It is stable in DMSO solutions but converts rapidly to much less active lumazine in aqueous media, the exposure time to which should be minimized as much as possible to maximize activity. All 5-OP-RU working solutions were diluted from the DMSO stocks with appropriate culture medium immediately (within 5 min) prior to the proliferation assay.

### Preparation of sIL-7Rα/IL-7 or sIL-7Rα/IL-2 mixture

Recombinant human IL-7 (R&D System) or IL-2 (PeproTech) at 2.5 ng/mL in MAIT cell Expansion Medium (see below) were incubated with sIL-7Rα (Biolegend) at varying amounts corresponding to the indicated molar ratios for 1 h at 37°C. PBMCs were then cultured in these media for further MAIT cell functional and proliferation assay as indicated.

### MAIT cells activation and proliferation assays

MAIT cells within bulk PBMCs, identified as Vα7.2^+^ CD161^hi^ CD3^+^ T cells, were activated using formaldehyde-fixed *E. coli* at the microbial dose of 1 for 24 h as previously described (65). Monensin (Golgi Stop, BD Biosciences) was added for the last 6 h of incubation. To determine the effect of sIL-7Rα on IL-7-dependent MAIT cell activation, cryopreserved PBMCs were thawed and rested for 48 h in a 37°C/5%CO_2_ incubator in ImmunoCult-XF T cell expansion medium supplemented with 8% (v/v) xeno-free CTS Immune Cell Serum Replacement (ThermoFisher Scientific), 50 μg/mL gentamicin (ThermoFisher Scientific), and 100 μg/mL normocin (Invivogen) (collectively: Expansion Medium). PBMCs were subsequently supplemented with IL-7 alone or in combination with sIL-7Rα as indicated for a further 24 h prior to analyses. In selected experiments, PBMCs were further stimulated with formaldehyde-fixed *E. coli* at the microbial dose of 0.1 for another 24 h.

For MAIT cell proliferation assay, MAIT cells were expanded as previously described (59, 66) with modifications. Briefly, cryopreserved PBMCs were thawed and stained using CTV cell proliferation dye as previously described (67), and rested for 48h in a 37°C/5%CO_2_ incubator in Expansion Medium. Subsequently, PBMCs were cultured with 100 pM 5-OP-RU with or without 2.5 ng/mL IL-7 or IL-2 alone, or with varying amounts of sIL-7Rα as indicated. No further media changes and cytokine supplementations were performed. Expansion and proliferation indices of MAIT cells, identified as MR1-OP-RU tetramer^+^ Vα7.2^+^ CD3^+^ T cells, were determined based on CTV dilution at day 5 post-stimulation using the FlowJo v. 10 software. Absolute MAIT cell numbers were calculated using Precision Count Beads (BioLegend) according to the manufacturer’s instruction.

### DNA extraction and IL-7Rα genotyping

DNA was extracted from neutrophils using DNeasy blood & tissue kit according to the manufacturer instruction (QIAGEN, Germany). 200ng of DNA from each patient was used to genotype the IL-7Rα gene. Six specific loci in both coding and non-coding regions of IL-7Rα gene (rs11567762, rs1494555, rs3194051, rs3822731, rs6897932 and rs987106) were genotyped using MassARRAY system through specific primer extension reactions coupled with matrix-assisted laser desorption/ionization time-of-flight (MALDI-TOF) for multiplexed genotyping (Sequenom MassARRAY iPLEX platform) performed at Australian Genome Research Facility (http://www.agrf.org.au/Genotyping-Platforms.html) or at China Sangon Biotech (https://www.sangon.com/zh/services_snp.html).

### IL-7Rα haplotype determination

Haplotypes of IL-7Rα were determined by genotyping six SNPs (haplotype-tagging SNP) of the IL-7Rα gene which collectively defined the four common haplotypes of the IL-7Rα gene as previously described (43). Briefly the rs1494555 SNP (T/C) tags haplotype 1, rs6897932 SNP (C/T) tags haplotype2, rs3822731 SNP (T/C) tags haplotype 3 and rs3194051 SNP (A/G) tags haplotype 4.

### Measurement of IL-7

IL-7 levels in plasma samples were measured using Quantikine HS ELISA kit following the manufacturer instruction (R&D Systems). This assay was performed on patient samples in the kinetics study. Briefly, plasma samples were diluted in 1 in 2 using the diluent provided. All samples were assayed in duplicate. Pre- and post-cART samples from the same patient were assayed in the same plate. The inter-assay and intra-assay CV for this assay were 16.3% (n=6) and 9.33% (n=6), respectively. The standard concentrations for this assay ranged from 0.25-16 pg/ml.

### Measurement of soluble IL-7Rα (sIL-7Rα)

sIL-7Rα levels in plasma samples were measured using an in-house ELISA method as previously described (42). Human IL-7Rα/Fc chimera extracellular domain immunoglobulin (R&D Systems) was used as standards and the concentration ranged from 0.625-40 ng/ml. Samples, standards, detection antibody and streptavidin-HRP were diluted using 1% BSA in PBS. The inter-assay and intra-assay CV precision for this assay were 20% (n=6) and 2.4% (n=6), respectively. All samples were assayed in duplicate.

### Statistical analysis

Statistical analyses were performed using Prism software v.9.4 (GraphPad). Data sets were first assessed for data normality distribution. Box and whisker plots show median, the 10^th^ to 90^th^ percentile, and the interquartile range. Statistically significant differences between samples were determined as appropriate using the unpaired t-test or Mann-Whitney’s test for unpaired samples. The Friedman test, one-way analysis of variance (ANOVA), or mixed-effects analysis followed by the appropriate *post-hoc* test as indicated was used to detect differences across multiple samples. Correlations were assessed using the Spearman rank correlation. Two-sided p-values < 0.05 were considered significant.

## Data availability statement

The raw data supporting the conclusions of this article will be made available by the authors, without undue reservation.

## Ethics statement

The studies involving human participants were reviewed and approved by The University Malaya Medical Centre’s ethics review board (MEC Ref: 896.32). The patients/participants provided their written informed consent to participate in this study.

## Author contributions

FH, MG, YZ, NZ, DH, AH, EL performed the experiments. FH, YZ, WS, EL analysed the data. EL designed the experiments. WS, LH, ZL, PQ, PL, AK, L-FW, JS, SL performed critical logistics and/or provided critical reagents. EL conceived the study. RR, EL managed the study. FH, RR, EL wrote the paper. WS, JKS revised the draft, which was approved by all authors. All authors contributed to the article and approved the submitted version.

## Funding

The research was supported by grants from Swedish Research Council Grant 2015-00174, Marie Skłodowska Curie Actions, Cofund, Project INCA 600398, the Jonas Söderquist Foundation for Virology and Immunology, the Petrus and Augusta Hedlund Foundation, Tsinghua Shenzhen International Graduate School Research Startup Funds (No. 01030100004), and the Shenzhen Pengcheng Peacock Program (to EL). The establishment of the clinical cohort and genotyping was supported by the High Impact Research grant (HIR/MOHE; H-20001-E000001) to RR, SL, and AK.

## Acknowledgments

The MR1 tetramer technology was developed jointly by Dr. James McCluskey, Dr. Jamie Rossjohn, and Dr. David Fairlie; and the material was produced by the NIH Tetramer Core Facility as permitted to be distributed by the University of Melbourne. The MR1 ligand 5-(2-oxopropylideneamino)-6-D-ribitylaminouracil (5-OP-RU) was kindly provided by Drs. David P. Fairlie and Jeffrey Y. W. Mak (University of Queensland, Australia) (62, 63).

## Conflict of interest

The authors declare that the research was conducted in the absence of any commercial or financial relationships that could be construed as a potential conflict of interest.

## Publisher’s note

All claims expressed in this article are solely those of the authors and do not necessarily represent those of their affiliated organizations, or those of the publisher, the editors and the reviewers. Any product that may be evaluated in this article, or claim that may be made by its manufacturer, is not guaranteed or endorsed by the publisher.

## Supplementary material

Supplementary Figure 1 Age distribution and MAIT cell levels in HIV-1-uninfected cohort. **(A)** Age distribution stratified by sex in HC carrying the *IL7RA* haplotype 2 vs non-haplotype 2. **(B)** MAIT cell levels in female and male participants of the healthy cohorts stratified by *IL7RA* haplotype. **(B)** Box and whisker plots show all data points, median, and the interquartile range. Statistical significance was determined using Mann-Whitney’s test. ns, not significant.

Supplementary Figure 2 Soluble IL-7Rα does not inhibit IL-2-mediated MAIT cell proliferation. **(A–G)** MR1-5-OP-RU tetramer^+^ Vα7.2^+^ MAIT cell **(A)** proliferation as determined by Ki-67 expression levels on Ki-67^+^ MAIT cells **(B**; *left panel* **, C)**, absolute MAIT cell numbers **(D)**, and proliferation **(E)** and expansion **(F)** indices calculated using CTV-dilution **(B**; *right panel* **)** following 5-days treatment of PBMC with 5-OP-RU, 5-OP-RU+IL-2, or 5-OP-RU+IL-2+sIL-7Rα (sIL-7Rα/IL-2 molar ratio 1:1) (N=5). **(G)** Dose-dependent effect of sIL-7Rα treatment on MAIT cells proliferation and expansion indices following 5-days treatment with IL-2 and 5-OP-RU (N=5). Expression levels for **(G)** were normalised to nil sIL-7Rα controls to minimise inherent donor-to-donor variability, with statistical tests were performed on raw data. Box and whisker plots show all data points, median, and the interquartile range, whereas data presented as line graphs with error bars represent the mean and standard error. Statistical significance was determined using the Friedman test followed by Dunn’s *post-hoc* test **(C, D)**, or repeated measure one-way ANOVA with Tukey’s *post-hoc* test **(E, F, G)**. ** p<0.01, * p<0.05, ns, not significant.
