# Supplementary figures and images for: IL7RA single nucleotide polymorphisms are associated with the size and function of the MAIT cell population in treated HIV-1 infection
Source: Front Immunol. 2022 Oct 20;13:985385. doi: 10.3389/fimmu.2022.985385 (PMC9632172; doi:10.3389/fimmu.2022.985385)

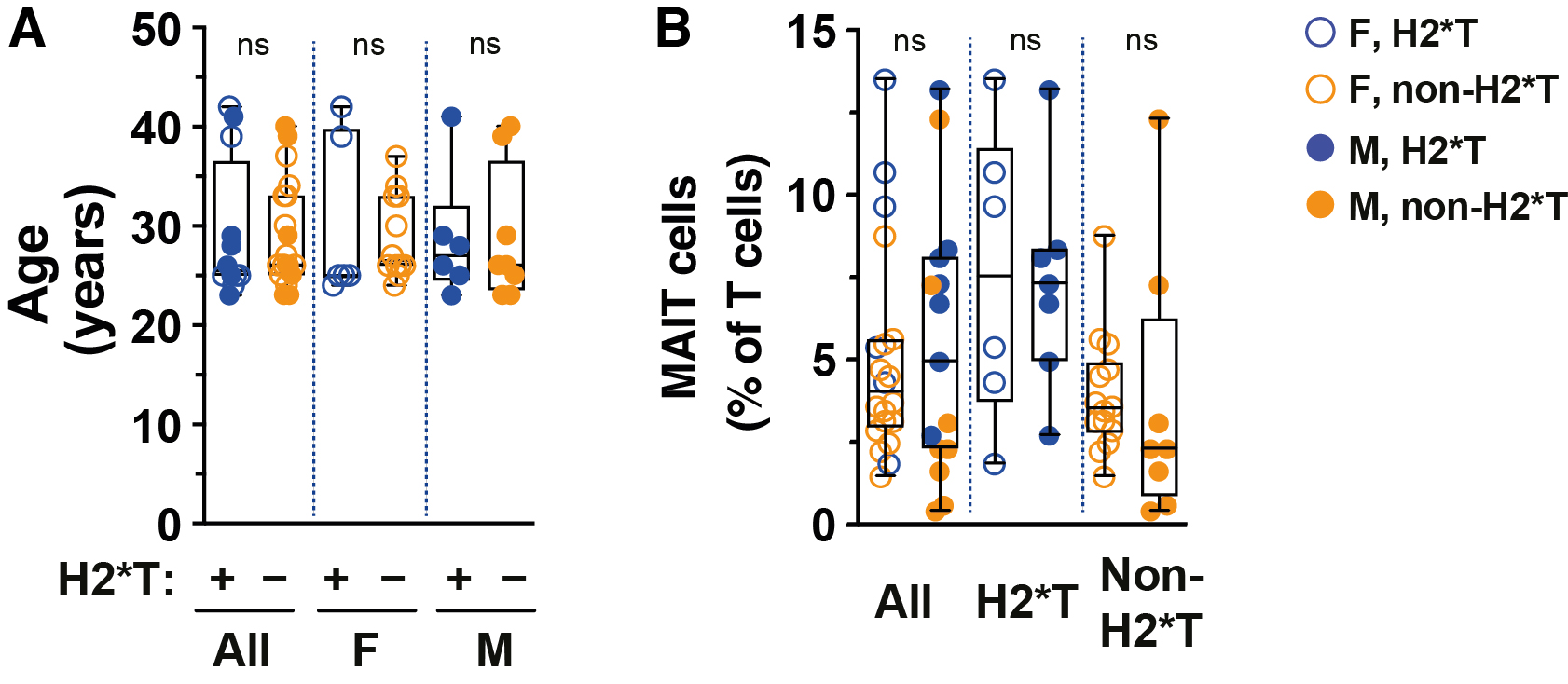

Supplement: Supplementary Figure 1 — Age distribution and MAIT cell levels in HIV-1-uninfected cohort. (A) Age distribution stratified by sex in HC carrying the IL7RA haplotype 2 vs non-haplotype 2. (B) MAIT cell levels in female and male participants of the healthy cohorts stratified by IL7RA haplotype. (B) Box and whisker plots show all data points, median, and the interquartile range. Statistical significance was determined using Mann-Whitney’s test. ns, not significant. [file Image_1.jpeg]

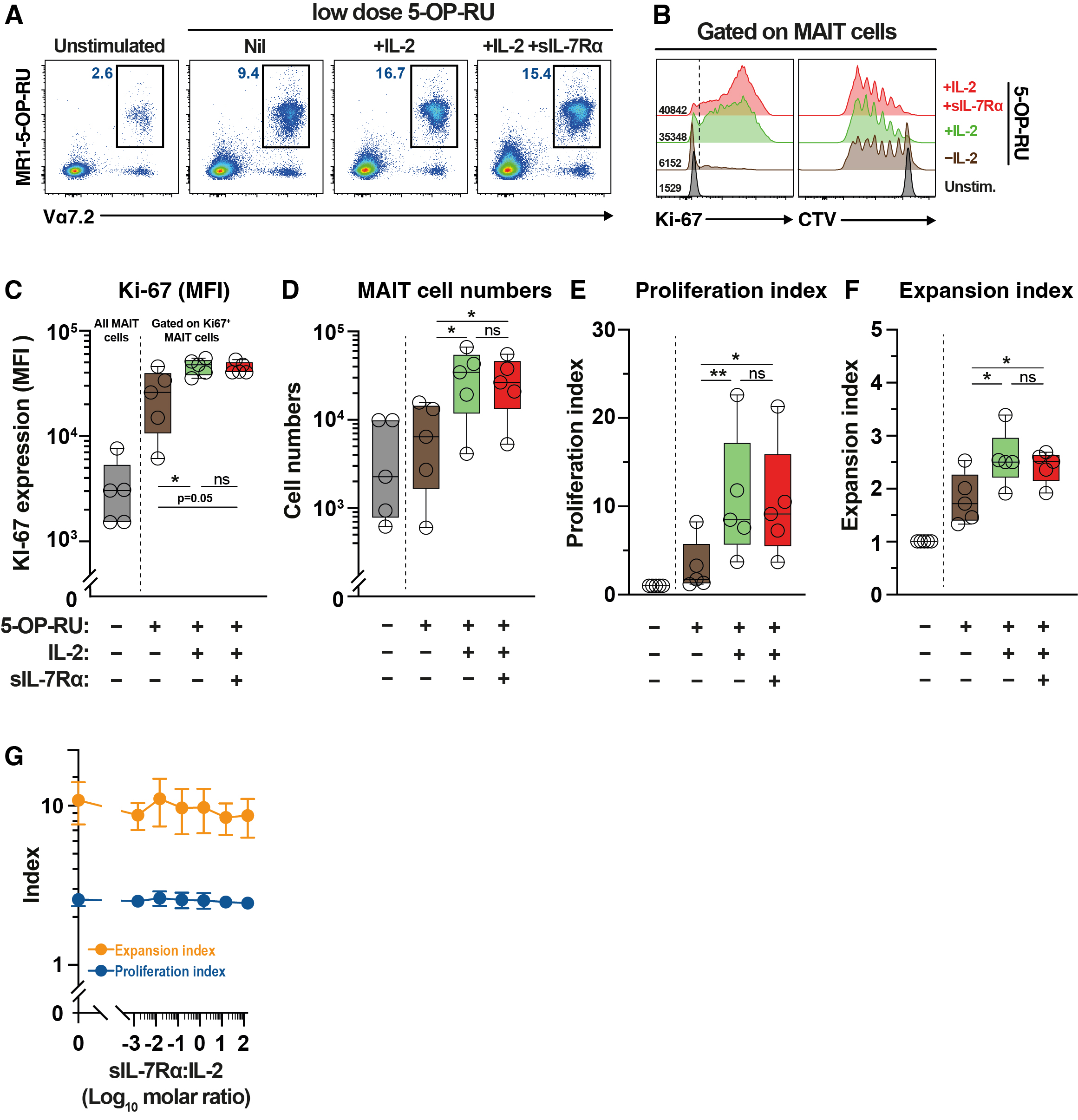

Supplement: Supplementary Figure 2 — Soluble IL-7Rα does not inhibit IL-2-mediated MAIT cell proliferation. (A–G) MR1-5-OP-RU tetramer+ Vα7.2+ MAIT cell (A) proliferation as determined by Ki-67 expression levels on Ki-67+ MAIT cells (B; left panel , C), absolute MAIT cell numbers (D), and proliferation (E) and expansion (F) indices calculated using CTV-dilution (B; right panel ) following 5-days treatment of PBMC with 5-OP-RU, 5-OP-RU+IL-2, or 5-OP-RU+IL-2+sIL-7Rα (sIL-7Rα/IL-2 molar ratio 1:1) (N=5). (G) Dose-dependent effect of sIL-7Rα treatment on MAIT cells proliferation and expansion indices following 5-days treatment with IL-2 and 5-OP-RU (N=5). Expression levels for (G) were normalised to nil sIL-7Rα controls to minimise inherent donor-to-donor variability, with statistical tests were performed on raw data. Box and whisker plots show all data points, median, and the interquartile range, whereas data presented as line graphs with error bars represent the mean and standard error. Statistical significance was determined using the Friedman test followed by Dunn’s post-hoc test (C, D), or repeated measure one-way ANOVA with Tukey’s post-hoc test (E, F, G). ** p<0.01, * p<0.05, ns, not significant. [file Image_2.jpeg]
