# Supplementary material for: IL7RA single nucleotide polymorphisms are associated with the size and function of the MAIT cell population in treated HIV-1 infection
Source: Front Immunol. 2022 Oct 20;13:985385. doi: 10.3389/fimmu.2022.985385 (PMC9632172; doi:10.3389/fimmu.2022.985385)
Supplement: Supplementary file 3 [file Table_1.docx]

**Supplementary Table 1**. **A schematic representation of the SNPs in the *IL-7RA* gene and *IL-7RA* haplotype** (adapted from (44))

| **Haplotypes** | **rs11567762** | **rs1494555** | **rs3194051** | **rs3822731** | **rs6897932** | **rs987106** |
| --- | --- | --- | --- | --- | --- | --- |
| **Haplotype 1** | G | C | A | T | C | T |
|  | A | C | A | T | C | T |
| **Haplotype 2** | G | T | A | T | T | A |
| **Haplotype 3** | G | T | A | C | C | T |
| **Haplotype 4** | G | T | G | T | C | A |

**Supplementary Table 2. Characteristics of the HIV-1-unifected healthy controls.**

|  | **Haplotype 2** | **Non-Haplotype 2** | ***p*-values** |
| --- | --- | --- | --- |
| N | 13 | 22 |  |
| Sex, F/M (N) | 6/7 | 14/8 | 0.48^+^ |
| Age (years) | 26 (23-42) | 26 (23-40) | 0.72^*^ |
| Age, F (years) | 25 (24-42) | 27 (24-37) | 0.53^*^ |
| Age, M (years) | 27 (23-41) | 26 (23-40) | 0.82^*^ |

All data show median and the range inside the brackets. Significance was determined using ^*^Mann-Whitney’s test and ^+^Fisher’s exact test. F, female; M, male.

**Supplementary Table 3. Spearman’s correlation between age and plasma sIL-7Rα or MAIT cell characteristics in HIV-1-unifected healthy controls.**

| **Spearman’s correlation** | **sIL-7Rα**  **(pg/mL)** | **MAIT cells**  **(% of T cells)** | **IFNγ^+^**  **(% of MAIT cells)** | **TNF^+^**  **(% of MAIT cells)** | **IFNγ^+^TNF^+^**  **(% of MAIT cells)** |
| --- | --- | --- | --- | --- | --- |
| Age | -0.06^ns^ | -0.03^ns^ | -0.19^ns^ | -0.04^ns^ | 0.43^ns^ |
| Age, F | 0.0^ns^ | 0.03^ns^ | -0.16^ns^ | -0.15^ns^ | 0.52^ns^ |
| Age, M | 0.02^ns^ | -0.17^ns^ | 0.01^ns^ | 0.32^ns^ | 0.35^ns^ |
| Age, H2*T | -0.68^ns^ | 0.19^ns^ | -0.44^ns^ | -0.63^ns^ | 0.63^ns^ |
| Age, non-H2*T | 0.03^ns^ | -0.34^ns^ | -0.09^ns^ | 0.23^ns^ | 0.44^ns^ |

All correlations were calculated using Spearman’s correlation. F, female; M, male; ns, non-significant (p>0.05); s, soluble.

**Supplementary Table 4. Clinical characteristics of the HIV-1-infected cohort.**

|  | **Haplotype 2** | **Non-Haplotype 2** | ***p*-values** |
| --- | --- | --- | --- |
| N | 15 | 24 |  |
| Age (years) | 45 (37-54) | 44 (33-55) | 0.35^#^ |
| Baseline VL (Log_10_ RNA copies/mL) | 5 (3.92-6.24) | 5 (3.77-5.86) | 0.97^*^ |
| Baseline CD4 (cells/μL) | 113 (8-314) | 76 (8-250) | 0.53^#^ |
| Current CD4 (cells/μL) | 562 (93-934) | 512 (124-937) | 0.84^#^ |
| Baseline CD8 (cells/μL) | 619 (187-1436) | 730 (146-2453) | 0.59^*^ |
| Age on ART initiation (years) | 37 (32-46) | 36 (22-49) | 0.5^#^ |
| ART duration (years) | 7 (3-12) | 7 (2-10) | 0.38^#^ |
| History of AIDS defining illness | 10 (66.7%) | 14 (58.3%) | 0.74^+^ |

All data show median and the range inside the brackets, except for the AIDS defining illness row where it shows the number of individuals and as a percentage of the sub-group with history of AIDS defining illness. Significance was determined using ^#^ unpaired t-test, ^*^Mann-Whitney’s test, and ^+^Fisher’s exact test.

**Supplementary Table 5. Spearman’s correlation between age and plasma sIL-7Rα or MAIT cell characteristics in HIV-1-infected cohort.**

| **Spearman’s correlation** | **sIL-7Rα**  **(pg/mL)** | **MAIT cells**  **(% of T cells)** | **IFNγ^+^**  **(% of MAIT cells)** | **TNF^+^**  **(% of MAIT cells)** | **IFNγ^+^TNF^+^**  **(% of MAIT cells)** |
| --- | --- | --- | --- | --- | --- |
| Age (all M) | -0.45^ns^ | -0.02^ns^ | 0.13^ns^ | 0.19^ns^ | 0.36^ns^ |
| Age, H2*T | -0.36^ns^ | 0.35^ns^ | 0.36^ns^ | -0.10^ns^ | 0.09^ns^ |
| Age, non-H2*T | -0.35^ns^ | -0.03^ns^ | 0.40^ns,^^ | 0.0^ns,^^ | -0.80^ns,^^ |

All correlations were calculated using Spearman’s correlation. M, male; ns, non-significant (p>0.05); s, soluble; ^^^, n<5.
